# Profile of adult intensive care units in Brazil: systematic review of observational studies

**DOI:** 10.5935/0103-507X.20210088

**Published:** 2021

**Authors:** Luciana Mara Meireles Aguiar, Gabriela de Sousa Martins, Renato Valduga, André Paz Gerez, Eduardo Cunha do Carmo, Katiane da Costa Cunha, Graziella França Bernardelli Cipriano, Marianne Lucena da Silva

**Affiliations:** 1 Secretaria de Estado e Saúde do Distrito Federal - Brasília (DF), Brazil.; 2 Universidade de Brasília - Brasília (DF), Brazil.; 3 Universidade do Estado do Pará - Belém (PA), Brazil.; 4 Universidade Federal de Jataí - Goiás (GO), Brazil.

**Keywords:** Critical care outcomes, Health services research, Epidemiology, Intensive care units, Brazil

## Abstract

**Objective:**

To identify the clinical and epidemiological profile of adult intensive care units in Brazil.

**Methods:**

A systematic review was performed using a comprehensive strategy to search PubMed®, Embase, SciELO, and the *Biblioteca Virtual em Saúde*. The eligibility criteria for this review were observational studies that described the epidemiological and/or clinical profile of critically ill patients admitted to Brazilian intensive care units and were published between 2007 and 2020.

**Results:**

From the 4,457 identified studies, 27 were eligible for this review, constituting an analysis of 113 intensive care units and a final sample of 75,280 individuals. There was a predominance of male and elderly patients. Cardiovascular diseases were the main cause of admission to the intensive care unit. The Acute Physiology and Chronic Health Evaluation II score was the most widely used disease severity assessment system. The length of stay and mortality in the intensive care unit varied widely between institutions.

**Conclusion:**

These results can help guide the planning and organization of intensive care units, providing support for decision-making and the implementation of interventions that ensure better quality patient care.

**Registration PROSPERO:** CRD4201911808.

## INTRODUCTION

Knowledge of the health conditions of a population, as well as its determinants, trends, and characteristics of the health/disease process, helps us plan actions and make strategic decisions, resulting in higher quality of care and better health services offered.^(^1^,^2^)^

However, translating research evidence into clinical practice is usually a slow and challenging process.^(^3^)^ In Brazil, great socioeconomic inequality and regional disparities are factors that influence this process.^(^4^)^ The complexity of the regionalization of health in the country is due to such characteristics as its continental dimensions, its number of potential users, its regional inequalities and diversities, the scope of the State’s role in health, and the multiplicity of agents (governmental and nongovernmental; public and private) involved in providing health care.^(^5^)^

Intensive care units (ICUs) are an essential component of modern medicine. Intensive care units are diverse, with substantial variation related to geographic location, patient demography, ICU size, disease severity, and availability of intensivism, further complicating the applicability of quality improvement initiatives.^(^6^)^ The census conducted by the *Associação de Medicina Intensiva Brasileira* (AMIB)^(^7^)^ in 2016, based on information from the National Registry of Health Establishments, indicated that in Brazil, there were 41,741 ICU beds, including in public, private, and philanthropic hospitals, and 27,709 beds were intended for adult patients in critical condition. In 2018, a survey conducted by the Federal Council of Medicine indicated that the number of ICU beds in Brazil was 44,253, and 49% were available for the Unified Health System (SUS - *Sistema Único de Saúde*).^(^8^)^ In addition, of the 5,570 Brazilian municipalities, ICU beds were available in only 532, with 53.4% of them in the Southeast region.^(^8^)^ This may lead to the need to travel between regions of the country to obtain these services.^(^9^)^ The Brazilian scenario has heterogeneity both in its extent and in its sociodemographic development, which can lead to unequal growth, with important implications for the distribution of goods and services, especially those related to health.^(^10^)^

In this context, it is important to identify the characteristics of Brazilian ICUs so that health professionals and managers can have information that will promote the planning, safety, and quality of care for critically ill patients. The present study aimed to characterize the clinical and epidemiological profile of adult ICUs in Brazil based on published data through a systematic review.

## METHODS

The studies were selected according to the Preferred Reporting Items for Systematic Reviews and Meta-Analyses (PRISMA) guidelines.^(^11^)^ The study protocol was registered in PROSPERO (www.crd.york.ac.uk/prospera/) under number CRD42019118081. Two independent authors initially evaluated the title and abstract. After the selection of potentially relevant studies, the full-text versions were independently analyzed by two researchers. Disagreements were resolved by discussion.

### Strategy for search and selection of studies

The potential studies going into this review were identified through a comprehensive strategy of searching the databases PubMed®, Embase, Scientific Electronic Library Online (SciELO), and *Biblioteca Virtual em Saúde* (BVS). A complementary search was performed on the reference lists of the selected articles to retrieve relevant publications.

The database searches were performed from August to December 2020, involving the cross-checking of descriptors selected in the medical subject heading (MeSH) terms of the National Library of Medicine of the United States. All terms were adapted for each database and combined using Boolean digits. The complete search strategy is shown in table 1.

The eligibility criteria for this review were observational studies published from 2007 to 2020 that aimed to describe the epidemiological and/or clinical profile of critically ill adult patients of both sexes, as well as the length and outcome of hospitalization in Brazilian ICUs. The studies were excluded for the following reasons: studies that selected a subgroup of patients with specific disease or clinical condition, randomized clinical trials or review articles, theses or dissertations, full text not available, abstracts and publications at conferences, and studies that used the same data sources as another included study.

**Table 1:** Detailed search strategy by database

Database	Research strategy
BVS	(“health profile” OR “health status” OR mortality OR demography OR epidemiology OR “epidemiological profile” OR “outcome measure” OR “Health level” OR “outcome studies” OR “outcomes research” OR “health service” OR “frequency” OR prevalence OR incidence)) AND (“intensive care unit” OR icu OR uti OR “critical care” OR “critical illness” OR “Critical care outcomes”)) AND (brazil OR brazil OR brazil OR “Latin America” OR “South America”))) AND (instance:”regional”) AND (limit:(“humans”) AND year_cluster :(“2013” OR “2014” OR “2012” OR “2015” OR “2010” OR “2011” OR “2008” OR “2016” OR “2009” OR “2007” OR “2017” OR “2018” OR “2019” OR “2020”))
PubMed®	((((“*health status*”[*MeSH Terms*] *OR* (“*demography*”[*MeSH Terms*] *OR* “*demography*”[*All Fields*]) *OR* ((“*epidemiology*”[*MeSH Terms*] *OR* “*epidemiology*” [*Subheading*] *OR* “*epidemiological*”[*All Fields*]) *OR profile*[*All Fields*])) *AND* ((“*intensive care units*”[*MeSH Terms*] *OR UTI*[*All Fields*] *OR CTI*[*All Fields*]) *OR ICU*[*All Fields*])) *AND* ((“*brazil*”[*MeSH Terms*] *OR* “*brazil*”[*All Fields*]) *OR* brasil[*All Fields*])) *NOT* ((“*infant, newborn*”[*MeSH Terms*] *OR* (“*infant*”[*All Fields*] *AND* “*newborn*”[*All Fields*]) *OR* “*newborn infant*”[*All Fields*] *OR* “*neonatal*”[*All Fields*]) *OR* (“*pediatrics*”[*MeSH Terms*] *OR* “*pediatrics*”[*All Fields*] *OR* “*pediatric*”[*All Fields*]) *OR* (“*child*”[*MeSH Terms*] *OR* “*child*”[*All Fields*] *OR* “*children*”[*All Fields*])) *AND* “*humans*”[*MeSH Terms*]
Embase	(‘*health status*’ *OR demography OR epidemiology OR* ‘*health level*’ *OR* ‘*health service*’) *AND* (‘*intensive care unit*’ *OR icu OR uti OR* ‘*critical care*’ *and OR* ‘*critical illness*’ *OR* ‘*critical care outcomes*’) *AND* (*Brazil OR Brazil OR Brazilian OR* ‘*Latin America*’ *OR* ‘*South America*’) *AND* [*2007-2020*]/*py*
SciELO	*AND* Profile “Intensive Care Units”

BVS - *Biblioteca Virtual em Saúde*; SciELO - Scientific Electronic Library Online.

### Data extraction and quality assessment

For the purposes of analysis and composition of the results, the following data were considered: study characteristics (design, sample size, institution profile, number of ICUs, Brazilian region, and state); sociodemographic aspects of the critical patient population treated in the ICUs (sex, age, race, education, marital status, and religion); and clinical characteristics (prognostic indices for assessment of disease severity upon admission to the ICU, origin of the patient as clinical or surgical, therapeutic interventions related to the use of invasive mechanical ventilation (IMV), vasoactive drugs and/or hemodialysis throughout the ICU stay, main causes of ICU admission, length of stay, and clinical outcome in the ICU as death or discharge).

The methodological quality and risk of bias of the included articles were evaluated by two researchers independently using the criteria of the Newcastle-Ottawa scale (NOS) and the Joanna Briggs Institute (JBI) Critical Appraisal Checklist for Analytical Cross-Sectional Studies, respectively. The JBI scale has nine questions to answer, divided between the participant domains (questions 1, 2, 4, and 9), measurement of results (questions 6 and 7), and statistics (questions 3, 5, and 8). A paper was classified as high quality when the methods were appropriate in all domains.^(^12^)^ The NOS is graded through a star system from 0 to 9, delimited into three domains (selection, comparability, and result). Higher grades represent better quality.^(^13^)^

### Data analysis

The variables were collected and tabulated in a spreadsheet to compose the results. Quantitative variables are reported as mean ± standard deviation or median (interquartile range). Categorical variables are given as absolute number (n) and frequency (%). All analyses were conducted using the Microsoft Excel 2013 descriptive statistics package.

## RESULTS

**Figure 1:**
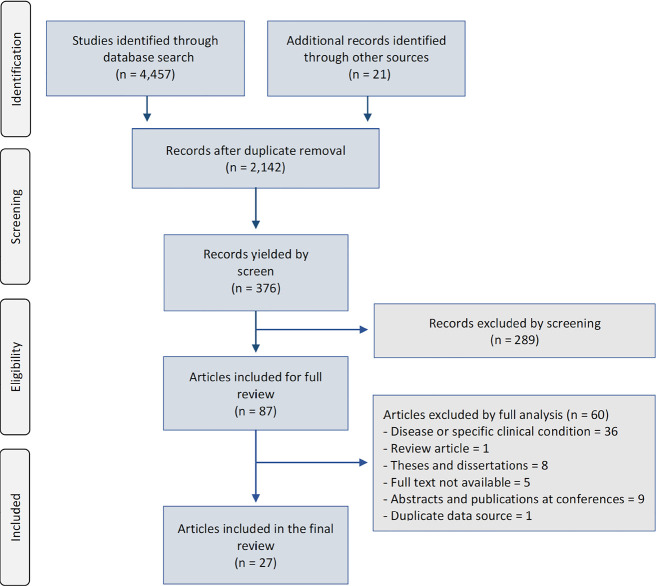
Flowchart of the review study.

The research strategy yielded a total of 4,478 studies. After removing duplicates and screening the titles and abstracts, 87 studies were selected for verification of the full text, of which 27 were eligible to be evaluated by this review (Figure 1).

### Characteristics of the studies

Of the 27 eligible studies (Table 2), 18 were descriptive, with a quantitative and retrospective approach,^(^15^,^17^-^20^,^24^,^26^-^28^,^30^-^32^,^34^-^39^)^ and seven were prospectively performed.^(^14^,^16^,^22^,^23^,^25^,^29^,^40^)^ Data from all studies were collected from patient records, sector record books, and computerized database systems.

**Figure 2:**
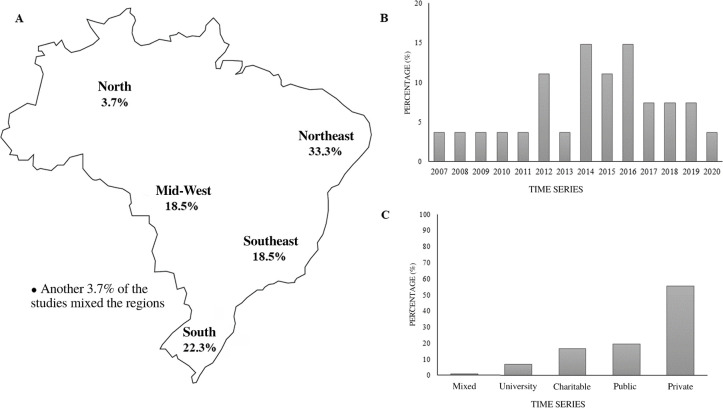
Overview of the origins of the studies included in the review. Percentage distribution by region (A), by historical series (B), and by the institutional profile of the intensive care unit (C).

In total, the studies investigated 113 ICUs, 63 of them private, 22 public, and the others philanthropic, university, or mixed institutions. They were most often located in the Northeast region (33.3%), followed by the South region (22.3%), Southeast region (18.5%), Central-West region (18.5%), and the North region (3.7%). One study was conducted in more than one region.^(^40^)^ Some 81.5% of the studies were published in 2012 or later, especially between 2014 and 2016, and they were conducted predominantly (52%) in private ICUs (Figure 2).

### Sociodemographic and clinical profile of patients in Brazilian intensive care units

The sample studied in this review was 75,280 individuals, with a predominance of males in 81% of the included studies. The age of participants monitored in the ICUs ranged from a minimum age of 12 years to a maximum of 104 years, with a predominance of mean ages greater than 50 years. There was a predominance of married individuals,^(^14^,^18^,^27^,^28^)^ the white and brown races,^(^14^,^19^,^28^)^ and low educational levels.^(^14^,^19^,^27^)^ Only one study identified religion, showing a predominance of Catholics (75.1%)^(^24^)^ (Table 3).

The mean length of stay in the ICU ranged from 1 to 23 days. The mortality rate reported in the studies ranged from 9.6% to 58%. Only eight studies^(^14^,^22^-^24^,^30^,^36^,^38^,^40^)^ indicated the severity of the patients by means of prognostic indices, Acute Physiology and Chronic Health Evaluation II (APACHE II) being the most used. Approximately 63% of the studies showed a predominance of clinical emergencies.

**Table 2:** Characteristics of the studies and institutions included

Study	Year	State	Study design	ICU (n)	Sample (n)
Acuña et al.^(^14^)^	2007	Acre	Prospective	1	79
Albuquerque et al.^(^15^)^	2017	Rio de Janeiro	Cross -sectional	1	573
Bezerra et al.^(^16^)^	2012	Paraíba	Prospective	1	140
Castro et al.^(^17^)^	2016	Goiás	Retrospective	3	2.579
Cruz et al.^(^18^)^	2019	Mato Grosso	Retrospective	1	86
El-Fakhouri et al.^(^19^)^	2016	São Paulo	Retrospective	1	2.022
Favarin et al.^(^20^)^	2012	Rio Grande do Sul	Retrospective	1	104
França et al.^(^21^)^	2013	Paraíba	Cross -sectional	1	102
Freitas et al.^(^22^)^	2010	Paraná	Prospectivo	4	146
Galvão et al.^(^23^)^	2019	Paraná	Prospectivo	1	3.711
Guia et al.^(^24^)^	2015	Federal District	Retrospective	1	189
Marques et al.^(^25^)^	2020	Sergipe	Prospective	1	43
Matias et al.^(^26^)^	2018	Mato Grosso	Retrospective	1	1.024
Melo et al.^(^27^)^	2014	São Paulo	Retrospective	1	479
Nascimento et al.^(^28^)^	2018	Paraíba	Retrospective	1	100
Nogueira et al.^(^29^)^	2009	Ceará	Prospective	1	157
Nogueira et al.^(^30^)^	2012	São Paulo	Retrospective	4	600
Pauletti et al.^(^31^)^	2017	Rio de Janeiro	Retrospective	2	975
Perão et al.^(^32^)^	2016	Santa Catarina	Retrospective	1	190
Del Painter et al.^(^33^)^	2015	Paraná	Cross -sectional	1	264
Queiroz et al.^(^34^)^	2013	Rio Grande do Norte	Retrospective	1	371
Rodriguez et al.^(^35^)^	2016	Santa Catarina	Retrospective	1	695
Silva et al.^(^36^)^	2008	Maranhão	Retrospective	1	297
Silva et al.^(^37^)^	2017	Bahia	Retrospective	1	284
Soares et al.^(^38^)^	2015	Mix*	Retrospective	78	59.693
Sousa et al.^(^39^)^	2014	Paraíba	Retrospective	1	310
Vieira et al.^(^40^)^	2012	Federal District	Prospective	1	67

ICU - intensive care unit.

* Bahia, Ceará, Federal District, Espírito Santo, Maranhão, Minas Gerais, Paraíba, Pernambuco, Rio de Janeiro, São Paulo, and Rio Grande do Sul.

ICU - intensive care unit.

**Table 3:** Sociodemographic characteristics of patients admitted to adult intensive care units in Brazil in 2007 - 2020

Study	Male sex (%)	Age (mean ± standard deviation)	Marital status (%)	Education (%)	Race (%)	Religion (%)
Acuña et al.(14)	67.1	53.3 ± 18.6	59.5 married	43 with <4 four years of study	59.5 white	- -
Albuquerque et al.^(^15^)^	53.0	66.5 ±19.4	- -	- -	- -	- -
Bezerra et al.^(^16^)^	49.6	65.8 ± 18.7	- -	- -	- -	- -
Castro et al.^(^17^)^	56.0	59.0	- -	- -	- -	- -
Cruz et al.^(^18^)^	43.1	39-59 years: 36.1%*	53.4 married	- -	- -	- -
El-Fakhouri et al.^(^19^)^	57.9	56.6 ± 19.18	- -	63.3 primary school	77.1 white	75.1 Catholic; 18.0 Protestant
Favarin et al.^(^20^)^	58.0	64.8 ± 5.6	- -	- -	- -	- -
França et al.^(^21^)^	55.9	53.2	- -	- -	- -	- -
Freitas et al.^(^22^)^	53.8	60.5 ± 19.2	- -	- -	- -	- -
Galvão et al.^(^23^)^	59.0	60.0	- -	- -	- -	- -
Guia et al.^(^24^)^	43.4	77.4 ± 10.9	- -	- -	- -	- -
Marques et al.^(^25^)^	55.8	68.0 ± 19.3	- -	- -	- -	- -
Matias et al.^(^26^)^	60.0	62-71 years: 33.2%*	- -	- -	- -	- -
Melo et al.^(^27^)^	64.9	49.0†	45.5 married	72.4 primary; 2.3 high school	- -	- -
Nascimento et al.^(^28^)^	58.0	58.8	48.0 married	- -	65.0 brown	- -
Nogueira et al.^(^29^)^	56.7	66.0	- -	- -	- -	- -
Nogueira et al.^(^30^)^	56.5	60.8 ± 18.7	- -	- -	- -	- -
Pauletti et al.^(^31^)^	58.4	- -	- -	- -	- -	- -
Perão et al.^(^32^)^	60.5	- -	- -	- -	- -	- -
Del Painter et al.^(^33^)^	- -	57.3 ± 19.8	- -	- -	- -	- -
Queiroz et al.^(^34^)^	51.4	64.8 ± 19.6	- -	- -	- -	- -
Rodriguez et al.^(^35^)^	61.6	50.0	- -	- -	- -	- -
Silva et al.^(^36^)^	44.6	- -	- -	- -	- -	- -
Silva et al.^(^37^)^	53.9	- -	- -	- -	- -	- -
Soares et al.^(^38^)^	49.9	62.0 ± 2.0	- -	- -	- -	- -
Sousa et al.^(^39^)^	54.8	- -	- -	- -	- -	- -
Vieira et al.^(^40^)^	58.2	49.3 ± 18.9	- -	- -	- -	- -

* Age expressed as frequency (%) by age group; † median.

Regarding the causes of ICU admission, there was a wide variety of described diseases, though cardiovascular disease (CVD) predominated in 66.7% of the included studies. The therapeutic interventions applied to critically ill patients have rarely been addressed in studies. The use of IMV was evaluated in eight studies,^(^14^,^16^,^23^-^25^,^31^,^38^,^40^)^ in which it was used in 10.7% to 74.3% of patients. The use of vasoactive drugs was addressed in five studies,^(^23^-^25^,^38^,^40^)^ and renal replacement therapy was addressed in only three studies^(^14^,^39^,^40^)^ (Table 4).

### Methodological quality of the selected studies

The quality of the studies was analyzed by the NOS (Table 5). The 27 included studies had a mean score of 3, a minimum of 1, and a maximum of 6 stars, which are considered bad scores because the highest score is 10. The risk of bias was assessed using the JBI checklist (Table 6).

## DISCUSSION

The present study identified the profile of Brazilian ICUs, characterizing them by the sex, age group, cause of ICU admission, length of stay, and ICU mortality of their patients as well as the most commonly used disease severity assessment system. These results are relevant because they allow us to understand the profile of both the user and the intensive care services and resources offered. Twelve of the 27 studies in this review reported that the ICU evaluated was the one primarily responsible for meeting the demand of the region, meaning that it received patients from other municipalities, which resulted in the overload of the service,^(^14^,^16^,^17^,^19^,^24^,^26^,^28^,^29^,^32^,^33^,^37^,^40^)^ the reallocation of more technological and human resources to these units, and the expansion of the network. This review found a predominance of male patients in the analyzed ICUs, which corroborates the findings of other studies.^(^41^)^ The factors that lead to the greater vulnerability of this population are the sociocultural construction of masculinity, neglect of risk control, poorer prevention of diseases and their complications, lower or late adherence to primary and secondary health services, inefficiency of specific policies, fear of serious illness, shame of exposing the body, absence of specialized units for human health, limited availability of public services, and more accidents and violence.^(^17^,^19^,^28^,^32^,^34^,^35^,^38^,^39^)^

**Table 4:** Clinical characteristics of patients admitted to adult intensive care units in Brazil in 2007-2020

Study	Main cause of ICU admission	Surgical profile (%)	ICU stay (days; mean ± standard deviation)	Prognostic indices (mean ± standard deviation)	Mortality in the ICU (%)	Therapeutic interventions (%)		
						MV	VAD	Hemodialysis
Acuña et al.^(^14^)^	Multiple-organ dysfunction syndrome	44.3	10.2 ± 9.6	APACHE II (18.4 ± 9.1)	38.0	51.9	- -	18.9
Albuquerque et al.^(^15^)^	Neurological diseases	42.0	10.7 ± 18.8	- -	26.0	- -	- -	- -
Bezerra et al.^(^16^)^	Cardiovascular diseases	- -	5.5 ± 5.6	- -	47.8	74.3	- -	- -
Castro et al.^(^17^)^	Cardiovascular diseases	37.0	7.6	- -	31.0	- -	- -	- -
Cruz et al.^(^18^)^	Cardiovascular diseases	34.9	≤ 10	- -	23.3	- -	- -	- -
El-Fakhouri et al.^(^19^)^	Cardiovascular diseases	- -	8.0 ± 10.7	- -	24.3	- -	- -	- -
Favarin et al.^(^20^)^	Infectious diseases	17.0	14.0	- -	50.0	- -	- -	- -
França et al.^(^21^)^	Cardiovascular diseases	- -	7.6	- -	48.0	- -	- -	- -
Freitas et al.^(^22^)^	- -	37.0	23.2 ± 23.7	APACHE II (20 ± 7.3)	58.2	- -	- -	- -
Galvão et al.^(^23^)^	Sepsis	38.7	16*	APACHE II (19)	32.2	10.7	7.1	- -
Guia et al.^(^24^)^	Respiratory diseases	- -	13.1 ± 6.1	APACHE II (1.6 ± 10.6)	38.6	56.6	50.8	- -
Marques et al.^(^25^)^	Cardiovascular diseases	44.9	10 ± 8	- -	- -	16.3	11.6	- -
Matias et al.^(^26^)^	Cardiovascular diseases	- -	- -	- -	23.5	- -	- -	- -
Melo et al.^(^27^)^	Cardiovascular and respiratory diseases	25.8	11.4	- -	35.3	- -	- -	- -
Nascimento et al.^(^28^)^	Cardiovascular diseases	32.0	10.6	- -	38.0	- -	- -	- -
Nogueira et al.^(^29^)^	Cardiovascular diseases	- -	- -	SAPS II (25.5)	54.1	- -	- -	- -
Nogueira et al.^(^30^)^	Cardiovascular diseases	36.0	9.0	- -	20.0	- -	- -	- -
Pauletti et al.^(^31^)^	Cardiovascular diseases	32.0	- -	- -	16.1	32.9	- -	- -
Perão et al.^(^32^)^	Cardiovascular diseases	40.0	- -	- -	25.1	- -	- -	- -
Del Painter et al.^(^33^)^	-	-	-	-	-	-	-	-
Queiroz et al.^(^34^)^	Cardiovascular diseases	10.0	3.4 ± 3.7	- -	30.2	- -	- -	- -
Rodriguez et al.^(^35^)^	Cardiovascular diseases	52.5	6.0	- -	20.4	- -	- -	- -
Silva et al.^(^36^)^	Neurological diseases	69.0	5.4	APACHE II (20.9)	18.3	- -	- -	- -
Silva et al.^(^37^)^	Cardiovascular diseases	- -	- -	- -	29.0	- -	- -	- -
Soares et al.^(^38^)^	Cardiovascular diseases	27.9	5 ± 9	SAPS III (43 ± 15)	9.6	15.2	12.8	2.8
Sousa et al.^(^39^)^	Cardiovascular diseases	12.6	- -	- -	46.5	- -	- -	- -
Vieira et al.^(^40^)^	Respiratory diseases	25.4	- -	APACHE II (25.8 ± 12.7)	50.7	73.1	58.2	50.7

ICU - intensive care unit; MV - mechanical ventilation; VAD - vasoactive drugs; APACHE II - Acute Physiology and Chronic Health Evaluation; SAPS - Simplified Acute Physiology Score.

* Median.

**Table 5:** Newcastle-Ottawa scale of the included studies

Author	Representativeness of the sample (*****)						Comparability (**)			Result (***)			Total
	1 (**)		2 (*)	3 (*)	4 (***)		1 (**)		1 (*****)			2 (*)	
	a (*)	b (*)	a (*)	a (*)	a (**)	b (*)	a (*)	b (*)	a (**)	b (**)	c (*)	a (*)	
Acuña et al.^(^14^)^						*						*	**
Albuquerque et al.^(^15^)^	*		*	*		*						*	*****
Bezerra et al.^(^16^)^	*		*			*						*	****
Castro et al.^(^17^)^						*							*
Cruz et al.^(^18^)^	*		*									*	***
El-Fakhouri et al.^(^19^)^	*		*			*							***
Favarin et al.^(^20^)^	*		*	*		*							****
França et al.^(^21^)^	*		*	*		*						*	*****
Freitas et al.^(^22^)^												*	*
Galvão et al.^(^23^)^	*		*	*		*						*	*****
Guia et al.^(^24^)^	*		*	*								*	****
Marques et al.^(^25^)^	*		*			*							***
Matias et al.^(^26^)^	*		*									*	***
Melo et al.^(^27^)^	*					*							**
Nascimento et al.^(^28^)^				*		*							**
Nogueira et al.^(^29^)^	*		*	*		*						*	*****
Nogueira et al.^(^30^)^	*		*	*		*						*	*****
Pauletti et al.^(^31^)^				*		*						*	***
Perão et al.^(^32^)^						*							*
Del Painter et al.^(^33^)^						*							*
Queiroz et al.^(^34^)^	*		*			*						*	****
Rodriguez et al.^(^35^)^	*		*	*		*						*	*****
Silva et al.^(^36^)^						*							*
Silva et al.^(^37^)^	*		*			*							***
Soares et al.^(^38^)^	*		*	*		*						*	*****
Sousa et al.^(^39^)^						*							*
Vieira et al.^(^40^)^												*	*

There was a predominance of patients older than 60 years admitted to the ICUs. Studies have estimated that 60% of ICU beds are occupied by patients older than 65 years, and the average length of stay of this group is 7 times greater than that of the younger population.^(^8^)^ The management of critically ill elderly patients is a complex issue that involves understanding the demographic changes of society and the physiology of aging. Decisions about the care of these patients in the ICU are based on criteria such as the reversibility of the causes of acute health deterioration, life expectancy, the baseline level of function of the patient, the severity of the disease, previous health status, and compliance with the patients’ and family members’ desire to perform invasive measures.^(^42^-^44^)^

In this review, the main cause of hospitalization in Brazilian ICUs was CVD. Brazil is among the countries with the highest mortality rate from CVD.^(^45^,^46^)^ Patients with these conditions require hospitalization in cardiac ICUs, coronary ICUs, or cardiothoracic surgery recovery units of to stabilize their clinical condition. In Brazil, the regional variations in the mortality rate from CVD can be attributed to specific profiles of the regions, which have different geographic characteristics, epidemiological characteristics, and organization of health services^(^47^,^48^)^

**Table 6:** Intrastudy risk of bias of the included studies according to the Joanna Briggs Institute Critical Appraisal Checklist for Analytical Cross-Sectional Studies

Author	Were the inclusion criteria in the sample clearly defined?	Were the study subjects and the environment described in detail?	Was exposure measured in a valid and reliable manner?	Were objective and standardized criteria used to measure the condition?	Have confounding factors been identified?	Were strategies established to deal with confounding factors?	Were the outcomes measured in a valid and reliable manner?	Was appropriate statistical analysis used?
Acuña et al.^(^14^)^	No	No	Yes	Yes	No	Not applicable	Yes	Yes
Albuquerque et al.^(^15^)^	Yes	Yes	Yes	Yes	No	Not applicable	Yes	Yes
Bezerra et al.^(^16^)^	Yes	Yes	Yes	No	No	Not applicable	Yes	Yes
Castro et al.^(^17^)^	No	Yes	Yes	Not clear	No	Not applicable	Not clear	Not clear
Cruz et al.^(^18^)^	Yes	Yes	Yes	Yes	No	Not applicable	Yes	No
El-Fakhouri et al.^(^19^)^	Yes	Yes	Yes	Yes	No	Not applicable	Yes	No
Favarin et al.^(^20^)^	Yes	Yes	Yes	Yes	No	Not applicable	Yes	No
França et al.^(^21^)^	Yes	Yes	Yes	Yes	No	Not applicable	Yes	Yes
Freitas et al.^(^22^)^	No	Yes	Yes	Yes	No	Not applicable	Yes	Yes
Galvão et al.^(^23^)^	Yes	Yes	Yes	Yes	No	Not applicable	Yes	Yes
Guia et al.^(^24^)^	Yes	Yes	Yes	Yes	No	Not applicable	Yes	Yes
Marques et al.^(^25^)^	Yes	Yes	No	Yes	No	Not applicable	Yes	Not clear
Matias et al.^(^26^)^	Yes	Yes	Yes	Yes	No	Not applicable	Yes	Yes
Melo et al.^(^27^)^	Yes	Yes	Yes	Yes	No	Not applicable	Not clear	Not clear
Nascimento et al.^(^28^)^	No	Yes	Yes	Yes	Yes	No	Yes	No
Nogueira et al.^(^29^)^	Yes	Yes	Yes	Yes	No	Not applicable	Yes	Yes
Nogueira et al.^(^30^)^	Yes	Yes	Yes	Yes	No	Not applicable	Yes	Yes
Pauletti et al.^(^31^)^	Yes	Yes	Yes	Yes	No	Not applicable	Yes	Yes
Perão et al.^(^32^)^	No	No	Not clear	Not clear	No	Not applicable	Not clear	Not clear
Del Painter et al.^(^33^)^	No	Yes	Yes	Yes	No	Not applicable	Yes	No
Queiroz et al.^(^34^)^	Yes	Yes	Yes	Yes	No	Not applicable	Yes	Yes
Rodriguez et al.^(^35^)^	Yes	Yes	Yes	Yes	No	Not applicable	Yes	No
Silva et al.^(^36^)^	No	No	Yes	Yes	No	Not applicable	Not clear	Not clear
Silva et al.^(^37^)^	No	No	Yes	Yes	No	Not applicable	Not clear	Not clear
Soares et al.^(^38^)^	Yes	Yes	Yes	Yes	No	Not applicable	Yes	Yes
Sousa et al.^(^39^)^	Yes	Yes	Yes	Not clear	No	Not applicable	Not clear	Not clear
Vieira et al.^(^40^)^	No	Yes	Yes	Yes	No	Yes	Yes	Yes

The ICU stay in this review ranged from 1 to 23 days. This measure is an important indicator of productivity and for planning care, as it reflects the peculiarities of the profile of each population.^(^19^,^49^)^ The patient’s stay in the ICU should be made as short as possible by reversing the acute condition to allow the patient to be transferred to another hospital unit of less complexity, avoiding the inappropriate use of the ICU.^(^16^,^27^,^39^)^ That is, in those with a high risk of death and limited medical care, interventions that painfully prolong the dying process should be avoided.^(^50^,^51^)^ In this context, the inclusion of palliative care in the ICU has been an important way to shorten ICU stays and lower overall health costs without hastening death, providing effective management of the pain and suffering of patients and their family members at the end of life.^(^52^)^

The studies in this review showed ICU mortality rates between 9.6% and 58%. Some factors associated with death were a longer stay (> 8 days), advanced age, greater disease severity (APACHE II > 20 points), comorbidities, decline in previous functional status, use of mechanical ventilation or vasoactive amines, acute renal failure, sepsis, and quality of care provided, which corroborates the findings of other national and international publications.^(^53^.^54^)^ Importantly, the mortality of critically ill patients admitted to the ICU may also be related to the natural evolution of the disease after the therapeutic possibilities have been exhausted.^(^15^)^

Few included studies used assessment systems for disease severity in the ICU. In recent decades, several scoring systems have been developed, among which APACHE II remains the most commonly used.^(^40^,^55^)^ The studies also rarely mentioned invasive therapies in the ICU. The use of MV, acute renal failure requiring renal replacement therapy, and the use of vasoactive drugs are factors associated with prolonged hospitalization and increased risk of morbidity and mortality.^(^56^)^ Knowing the therapeutic profile of ICUs is essential for the management of critical patients and the clinical and strategic decision-making of a healthcare unit.

This study has some strengths. It is the first systematic review to identify the profile of Brazilian ICUs in general based on published data, including studies from all regions of the country, with different kinds of institutions and a large final sample, which improves the representativeness of this study. Some of the results of this study agree with those of international studies. In the future, studies with greater methodological rigor and homogeneity of information should be done to allow meta-analyses to be run on their data, which would contribute to the consolidation of the national literature focused on high-complexity care.

This review also has some limitations. Observational studies are more vulnerable to methodological problems, which precluded a systematic review with meta-analysis. There was the possibility of publication bias: given our objective of delivering a broad and general characterization of ICUs, it is possible that some studies in specific populations did not meet the selection criteria for this review. Even so, to minimize the occurrence of this bias and gather as many results as we could, the literature search was broad, including in national and international scientific databases. It is also noteworthy that most of the included publications retrospectively profiled their ICUs, which could bring some information bias. Our evaluation of the quality of the studies highlighted methodological deficiencies.

## CONCLUSION

This systematic review on the profile of Brazilian intensive care units indicated that a growing number of studies have been conducted in different Brazilian regions in recent years, especially in public and general intensive care units covering all clinical specialties. Regarding the profile of these units, there was a predominance of male patients with a mean age greater than 50 years and elderly patients. Cardiovascular disease was the main cause of hospitalization in these intensive care units. The length of stay and mortality varied widely between institutions, depending on factors such as severity profile and region of residence of the patients. APACHE II is the disease severity assessment system most commonly used in Brazilian intensive care units, and most patients come from clinical emergency units. Few studies have investigated the sociodemographic characteristics or therapeutic interventions in intensive care units, which will be important to cover in new studies.

These results can help guide the planning and organization of intensive care units, both in the management of institutions and in regard to clinical practice, as they can support decision-making and the implementation of interventions to ensure better quality of patient care. We suggest conducting studies that better describe Brazilian intensive care units, using more rigorous methodological criteria and ensuring a higher quality of publications.
